# Atypical Bilateral Brain Synchronization in the Early Stage of Human Voice Auditory Processing in Young Children with Autism

**DOI:** 10.1371/journal.pone.0153077

**Published:** 2016-04-13

**Authors:** Toshiharu Kurita, Mitsuru Kikuchi, Yuko Yoshimura, Hirotoshi Hiraishi, Chiaki Hasegawa, Tetsuya Takahashi, Tetsu Hirosawa, Naoki Furutani, Haruhiro Higashida, Takashi Ikeda, Kouhei Mutou, Minoru Asada, Yoshio Minabe

**Affiliations:** 1 Department of Psychiatry and Neurobiology, Graduate School of Medical Science, Kanazawa University, Kanazawa, 920–8640, Japan; 2 Research Center for Child Mental Development, Kanazawa University, Kanazawa, 920–8640, Japan; 3 Department of Adaptive Machine Systems, Graduate School of Engineering, Osaka University, Suita, Osaka, 565–0871, Japan; University of Bern, SWITZERLAND

## Abstract

Autism spectrum disorder (ASD) has been postulated to involve impaired neuronal cooperation in large-scale neural networks, including cortico-cortical interhemispheric circuitry. In the context of ASD, alterations in both peripheral and central auditory processes have also attracted a great deal of interest because these changes appear to represent pathophysiological processes; therefore, many prior studies have focused on atypical auditory responses in ASD. The auditory evoked field (AEF), recorded by magnetoencephalography, and the synchronization of these processes between right and left hemispheres was recently suggested to reflect various cognitive abilities in children. However, to date, no previous study has focused on AEF synchronization in ASD subjects. To assess global coordination across spatially distributed brain regions, the analysis of Omega complexity from multichannel neurophysiological data was proposed. Using Omega complexity analysis, we investigated the global coordination of AEFs in 3–8-year-old typically developing (TD) children (n = 50) and children with ASD (n = 50) in 50-ms time-windows. Children with ASD displayed significantly higher Omega complexities compared with TD children in the time-window of 0–50 ms, suggesting lower whole brain synchronization in the early stage of the P1m component. When we analyzed the left and right hemispheres separately, no significant differences in any time-windows were observed. These results suggest lower right-left hemispheric synchronization in children with ASD compared with TD children. Our study provides new evidence of aberrant neural synchronization in young children with ASD by investigating auditory evoked neural responses to the human voice.

## Introduction

Autism spectrum disorders (ASD) appear in infancy or early childhood, causing delays or impairments in social interaction, communication, and a restricted range of interests. Altered auditory processing systems in children with ASD have been studied as neurological correlates of some phenotypes in ASD, such as inhibited language acquisition [1–8] and auditory hypersensitivity [9–11], and previous studies that have focused on auditory responses of the brain have also demonstrated altered cerebral laterality [2,5,6] and regional connectivity [12] in children with ASD. These studies have indicated alterations in cortical auditory processes in ASD [1,2,4,5,9,10], whereas increased rates of brain stem or peripheral hearing dysfunction have also been reported in the ASD population [13–20].

Magnetoencephalography (MEG) is a non-invasive neuroimaging technique that provides measures of cortical neural activities at a millisecond time scale. In the last decade, MEG has emerged as an important investigatory tool in neurodevelopmental studies because MEG has advantages over other neuroimaging techniques for young children, including safety, fewer constraints and less environmental noise. Using MEG, many recent studies have observed aberrant brain activities in children with ASD [1–10,21–29] by investigating brain connectivity [26,28–30] or atypical brain responses to auditory stimuli [1–10,21–25].

**Fig 1:**
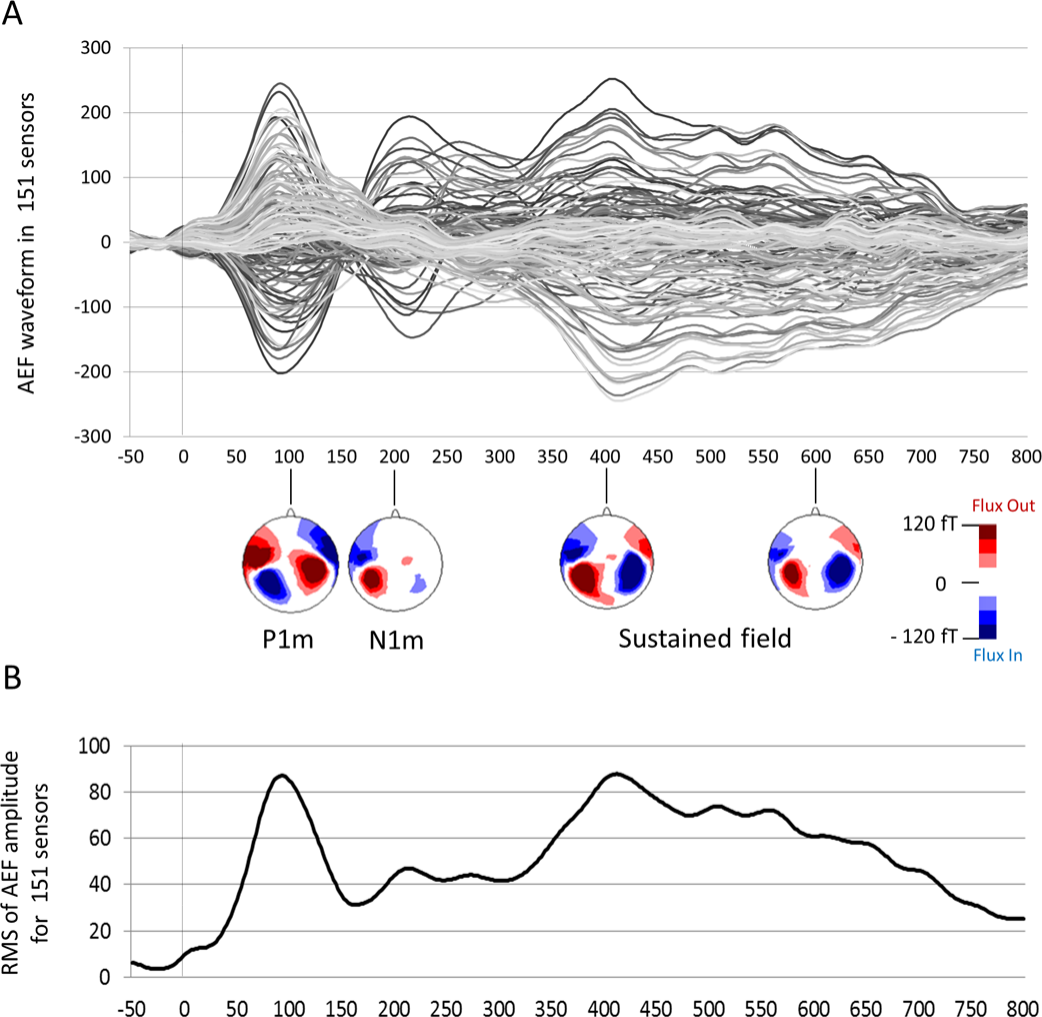
**Waveform of the AEF in 151 sensors (A) and the RMS for all sensors (B).** The data from one representative child aged 97 months is shown. Both the AEF waveform (A) and their RMS (B) demonstrated several identifiable peaks (i.e., P1m, N1m and sustained field). The first earliest cortical component of the AEF is P1m, which is a prominent component in 1- to 10-year-old children. The second earliest cortical component of the AEF is N1m. RMS, root mean square. AEF, auditory evoked field.

Auditory evoked field (AEF) is a brain response to auditory stimuli recorded by MEG and the equivalent of auditory evoked potential recorded by electroencephalography (EEG). AEFs recorded by MEG can clearly and easily distinguish bilateral brain responses evoked by binaural auditory stimuli. Thus, any unilateral abnormality or hemispheric difference can be accurately investigated by MEG [2]. The earliest cortical component of AEFs (i.e., P1m shown in Fig 1) is a prominent component in 1- to 10-year-old children [23,31–33]. In previous MEG studies, this component has been alternatively labeled M50, P50m or P100m. To avoid confusion, we call this component “P1m” in this study. The second earliest cortical component of AEFs is N1m. In previous MEG studies, this component has been alternatively labeled M100 or N100m; we here call this component “N1m.” The main sources of the P1m and N1m are the auditory cortex and association cortices, and abnormalities in AEF components have been reported in patients with ASD [1–10,21–25]. Although many prior studies have focused on the aberrant intensity or latency of AEF components in children with ASD, no investigations have focused on the bilateral synchronization of AEF components in children with ASD.

Reports have indicated that in addition to alterations in cortical auditory processes, the ASD population exhibits increased rates of brain stem or peripheral hearing dysfunction [13–20]. Dysfunctions in both peripheral and central auditory processing also distort the cortical bilateral synchronization of AEF components. The main aim of this study was to investigate the nature of bilateral brain synchronization of AEFs in TD children and children with ASD. In the present study, we employed a child custom-sized MEG system for AEF measurements and Omega complexity analysis [34,35] to estimate the degree of whole brain synchronization.

## Materials and Methods

### Participants

The clinical group consisted of 50 children with ASD (39 males, 11 females), aged 38–92 months, and recruited from the Kanazawa University Hospital and prefectural hospitals in Toyama. Children were diagnosed by a clinical psychiatrist and a clinical psychologist with more than 5 years of experience in ASD using the Autism Diagnostic Observational Schedule-Generic (ADOS) [36], the Diagnostic Interview for Social and Communication Disorders (DISCO) [37], the Kaufman Assessment Battery for Children (K-ABC) [38], and DSM-IV [39] criteria at the time of MEG and data acquisition. All ASD children included in this study fulfilled the diagnosis of childhood autism (n = 33), atypical autism (n = 9) or Asperger’s syndrome (n = 8) with DISCO. The controls were 50 typically developing children (39 males, 11 females) aged 36–97 months with no reported behavioral or language problems. The control children were approximately age-matched to the subjects with ASD. All typically developing children were native Japanese and had no prior or current developmental, learning, or behavioral problems. All participants had normal hearing ability according to available medical records. The dominant handedness of each participant was determined based on his or her preference for handling a spoon (TD children: right = 45, left = 4, both = 1; ASD children: right = 41, left = 2, both = 7). As shown in Table 1, the two groups were matched in chronological age and had similar K-ABC mental processing scale scores. Parents agreed to their child’s participation in the study with full knowledge of the experimental nature of the research. All participants were the same sample as in our previous study [28]. The raw scores of the K-ABC subtests in the TD children and the children with ASD are shown in S1 Fig. Based on the children’s assent, written informed consent was obtained prior to participation. The Ethics Committee of Kanazawa University Hospital approved the methods, and all procedures were performed in accordance with the Declaration of Helsinki.

**Table 1:** Demographic characteristics of the study participants.

Group	ASD children	TD children	*t* test
Number of subjects	50	50	
Age (range)	66.7 months (38 – 92)	66.8 months (36 – 97)	n.s.
Gender (M/F)	39/11	39/11	
K-ABC mental processing scale (± SD)	96.8 (± 22.5)	98.4 (± 14.1)	n.s.

K-ABC, Kaufman Assessment Battery for Children; TD, typically developing; ASD, Autism Spectrum Disorder; n.s., no significant difference (i.e., unpaired *t* test between two groups, *P* > 0.05).

### Magnetoencephalography recordings

The conditions used were similar to those detailed in our previous study [2,40,41]. The MEG data were recorded using a 151-channel SQUID (Superconducting Quantum Interference Device) whole-head coaxial gradiometer MEG system for children (PQ 1151R; Yokogawa/KIT, Kanazawa, Japan) in a magnetically shielded room (Daido Steel, Nagoya, Japan) that was installed at the MEG Centre of Yokogawa Electric Corporation (Kanazawa, Japan). The custom-sized MEG system facilitates the measurement of brain responses in young children, which would otherwise be difficult to measure using conventional adult-sized MEG systems. The child MEG system ensures that sensors are easily and effectively positioned within the range of the brain and that head movement is constrained [42]. An examiner remained in the room to encourage the children and prevent movement throughout the analysis. Stimuli were presented while the children lay in the supine position on the bed and viewed silent video programs projected onto a screen.

### Auditory evoked field stimuli and procedures

**Fig 2:**
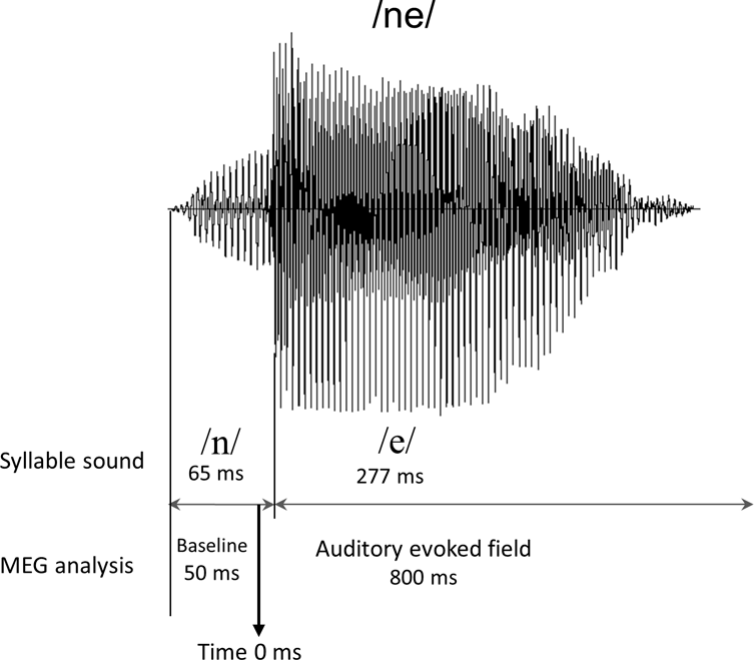
Sound waveform of the /ne/ voice stimulus. The total time of a /ne/ stimulus was approximately 342 ms; the time length of the consonant /n/ was approximately 65 ms; and the time length of the post consonantal vowel sound /e/ was approximately 277 ms. The time point of 50 ms after stimulation onset was defined as 0 ms, and the time-window of -50–0 ms was used as baseline period.

MEG recordings were obtained from all participants during auditory syllable sound stimulation that consisted of the Japanese syllable /ne/ [40]. We employed this syllable because /ne/ is one of the Japanese final sentence particles that carry prosodic information [43,44]. The syllable /ne/ is often used in Japanese mother—child conversations and expresses the speaker’s request for acknowledgement or empathy from the listener [45,46]. In the present study, we used typical oddball sequences consisting of standard stimuli at a rate of 83% (456 times) and deviant stimuli at a rate of 17% (90 times). In the standard stimulus, /ne/ was pronounced with a steady pitch contour, whereas in the deviant condition, /ne/ was presented with a falling pitch. Eventually, we adopted standard stimuli for subsequent Omega complexity analysis because a sufficient number of periods (i.e., 200 periods) were necessary for averaging to yield clear AEF waveforms after the rejection of artifacts in all children. A female native Japanese speaker produced the /ne/ sounds, which were presented using a condenser microphone (NT1-A; Rode, Silverwater, NSW, Australia) on a personal computer. As shown in Fig 2, the duration of the stimulus was 342 ms, and the duration of the consonant /n/ was 65 ms. In this study, we defined the time point of 50 ms after stimulation onset as 0 ms. The interstimulus interval (ISI) was 818 ms. Both stimuli had a level of approximately 65 dB (A-weighted) against a background noise of 43 dB, which was measured with an integrating sound level meter (LY20; Yokogawa, Tokyo, Japan). The stimulus was presented to participants binaurally through a hole in the MEG chamber using speakers (HK195 Speakers; Harman Kardon, Stamford, CT) that were placed outside of the shielded room. The duration of the recording was 12 minutes.

### AEF acquisition and analysis

The participant’s head was placed in a whole-head Dewar that contained 151 concentrically arranged magnetic sensors. The MEG data were acquired at a sampling rate of 1000 Hz, filtered using a 200 Hz low-pass filter and resampled at 500 Hz. The time series obtained started from the onset of the syllable stimulus at -50 ms and continued to 800 ms, and subsequent 200 segments (for standard stimuli) were averaged for each of the sensors after baseline correction (-50 to 0 ms) (Fig 1A). Segments that were contaminated with artifacts (eye-blink, and eye and body movements, typically more than ± 4 pT) were excluded from the analysis.

### Root mean squares in AEF for the whole head

To investigate the magnitude of AEFs in every time-window of 50 milliseconds, root mean squares (RMS) were calculated from the magnetic strength recorded by 151 sensors for each time-window. RMSs of TD children and children with ASD were compared with unpaired two-tailed *t-*tests for each time window. In the present study, we focused on 16 time windows (i.e., 0–50, 50–100, 100–150, 150–200, 200–250, 250–300, 300–350, 350–400, 400–450, 450–500, 500–550, 550–600, 600–650, 650–700, 700–750, and 750–800 ms). To adjust the significance level for the 16 time-windows, we used the Bonferroni correction and defined statistical significance as P < 0.0031.

### Root mean squares in AEF for the left and right hemispheres

**Fig 3:**
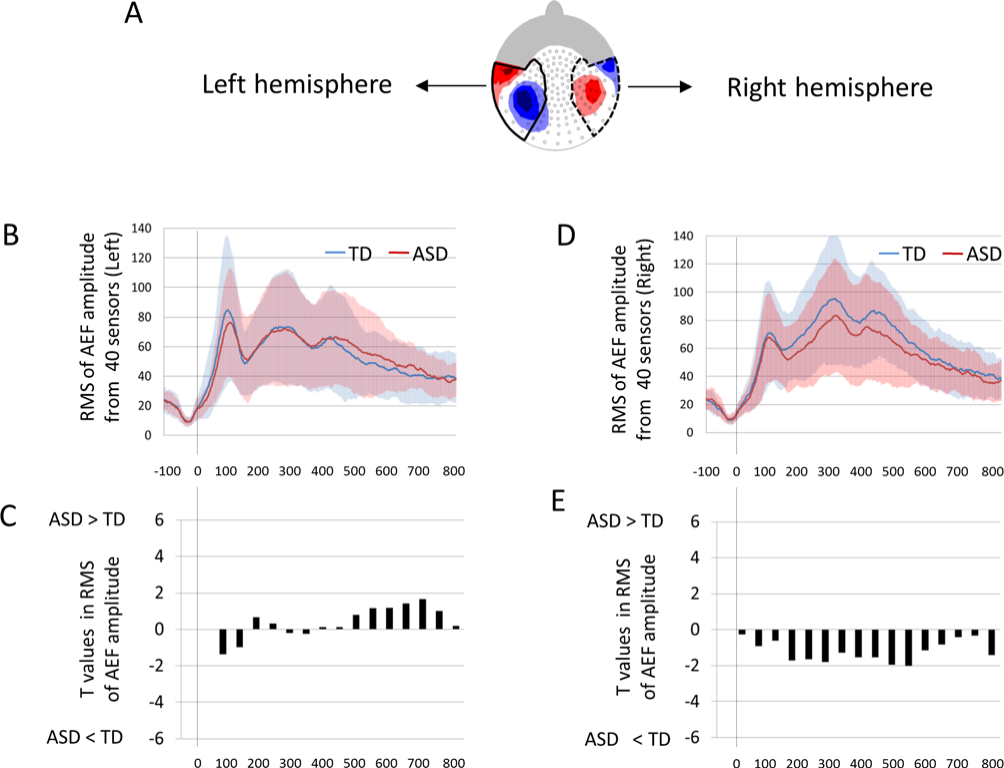
The magnitude of AEFs for the left and right hemispheres in TD and ASD children. (A) Selected 40 sensors that cover the AEF for each hemisphere. Red areas indicate magnetic field flux-out, and blue areas indicate magnetic field flux-in for P1m. RMSs were calculated in 40 sensors corresponding to the left (B) and right (D) hemisphere. The RMSs of TD children and children with ASD were compared with unpaired, two-tailed *t*-tests for 16 time windows in the left (C) and right (E) hemisphere. No significant differences were observed in any time-windows. RMS, root mean square. AEF, auditory evoked field. TD, typically developing. ASD, autism spectrum disorder. Faint colored zones represent 1 standard deviation.

To investigate the magnitude of AEFs in the left and right hemispheres, RMSs were also calculated from 40 sensors corresponding to both the left and right hemisphere (Fig 3A). RMSs of TD children and children with ASD were compared with unpaired two-tailed *t*-tests for 16 time windows. To adjust the significance level for multiple time-windows, we used the Bonferroni correction and defined statistical significance as P < 0.0031 for both the left and right hemisphere.

### AEF topography

As a complementary analysis, unpaired two-tailed *t*-tests were used to compare the magnitudes of AEFs in TD children and children with ASD for each of 151 sensors in each of 16 time windows. To adjust the significance level for these 16 time windows, we used the Bonferroni correction and defined a statistical significance threshold of P < 0.0031.

### Omega complexity in AEFs for the whole head

Off-line analysis of the MEG data was performed with the Brain Vision analyzer (Brain Products GmbH, Gilching, Germany) and Matlab (MathWorks, Natick MA). We employed Omega complexity analysis [34,35] to estimate the degree of whole brain synchronization during auditory evoked responses in every 50-ms time-window. Omega complexity is a single-value measure of the complexity of multichannel brain magnetic field data (in the following, K denotes the number of channels). The multichannel MEG data are viewed as a series of momentary maps. The trajectory of these maps over time forms an object in K-dimensional state space. Omega complexity evaluates the complexity of the data in the state space by examining the shape of this object, i.e., its extension along its principal axes. The computation of Omega complexity is based on a decomposition of the data into spatial principal components. In the present study, using 151-sensor data, a symmetrical 151 × 151 covariance matrix for all pairs of sensor for each time window was constructed. Principal component analysis of this covariance matrix yielded eigenvectors and eigenvalues (λ). The former represent the directions of principal axes in state space, and the latter represent the proportions of contribution of the respective components to the total variance. In the sense of state space representation, the vector of a principal component represents the spatial distribution, i.e., its topography, so that the respective eigenvalue represents the contribution of the topography to the total variance. To assess the relative contributions, the eigenvalues were normalized to the unit sum. The value of omega was calculated using the following equation:

$$Omega\;=\text{exp}\{\;-\sum_{i=1}^{k}\;\lambda_{i}^{\;\prime }\cdot \text{log}(\;\lambda_{i}^{\;\prime })\}\;,$$

in which λ′_i_ is the normalized eigenvalue of the i-th principal component.

Omega thus assesses the shape of the distribution of the spectrum of eigenvalues. An omega of 1 indicates a degenerate spectrum of eigenvalues, i.e., maximal synchronization of the signals at all locations. The highest complexity, omega = K, indicates a uniform distribution of eigenvalues, i.e., no correlation between any of the signals.

To estimate the degree of whole brain synchronization in AEFs in every 50-ms time-window, Omega complexity was log-10 transformed to obtain a normal distribution. The omega complexities of TD children and children with ASD were compared with unpaired two-tailed *t*-tests for each time window. To adjust the significance level for multiple time-windows, we used Bonferroni correction and defined statistical significance as P < 0.0031 for the 16 time-based comparisons.

If there was a significant difference in Omega complexity between the two groups (i.e., TD and ASD) even after the alpha level was adjusted, a complementary analysis was added to consider the three distinct subtypes of ASD (i.e., Asperger syndrome, and atypical and typical autism). One-way ANOVA of the four groups (i.e., TD, Asperger syndrome, and atypical and typical autism) was performed. Statistical significance was defined as P < 0.05. If there was a significant group effect in Omega complexity, a post-hoc t-test (unpaired) was performed, in which a Bonferroni correction was used. We defined statistical significance as P < 0.0083 for post-hoc comparisons (6 comparisons).

### Omega complexity in AEFs for the left and right hemispheres

To estimate the degree of brain synchronization in AEFs in the left and right hemispheres, Omega complexity was also calculated from 40 sensors corresponding to the left and right hemispheres (Fig 3A). The log-10 transformed Omega complexities of TD children and children with ASD were compared with unpaired two-tailed *t*-tests for the 16 time windows. To adjust the significance levels for multiple time-windows, we used the Bonferroni correction and defined statistical significance as P < 0.0031 for both the left and right hemispheres.

### Effects of age and cognitive ability on Omega complexity

To investigate the effects of age and cognitive ability (as assessed using the K-ABC mental processing scale) on the log-transformed Omega complexity, we calculated Pearson correlation coefficients between the transformed Omega complexity and each of these two factors. To adjust the significance level for the 16 examined time windows, we used the Bonferroni correction and defined a statistical significance threshold of P < 0.0031 for TD children and children with ASD.

## Results

### Root mean squares in AEFs for the whole head

**Fig 4:**
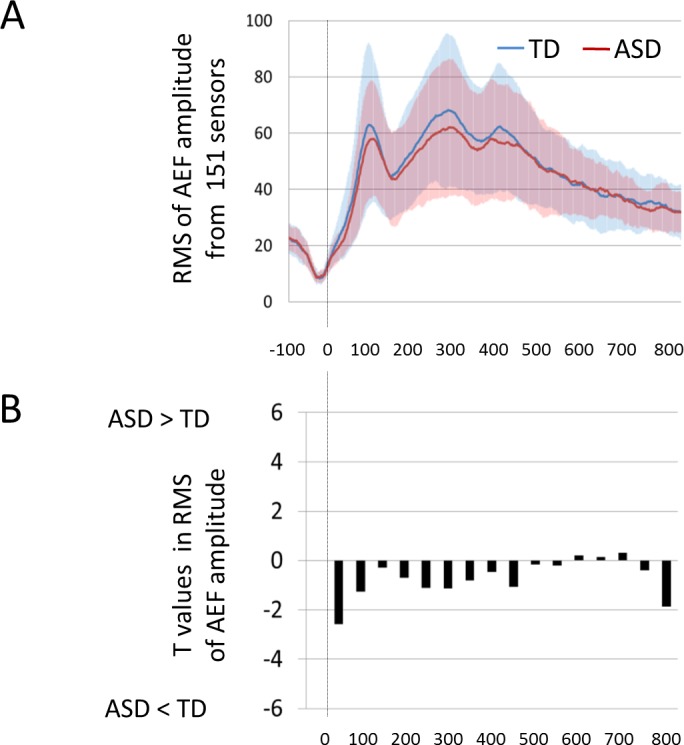
The magnitude of the AEF for the whole head in TD and ASD children. (A) RMSs were calculated by 151 sensors (whole head) in TD and ASD children. (B) For each time-window of 50 milliseconds, RMSs were compared between TD and ASD children with unpaired two-tailed *t*-tests for each time window. No significant differences were observed in any time-windows. RMS, root mean square. AEF, auditory evoked field. TD, typically developing. ASD, autism spectrum disorder. Faint colored zones represent 1 standard deviation.

As shown in Fig 4, although children with ASD tended to display lower RMSs compared with TD children during 0–500 ms, no significant differences were observed in any time-windows (P > 0.0031).

### Root mean squares in AEFs for the left and right hemisphere

In the left hemisphere, as shown in Fig 4B and 4C, although children with ASD tended to display higher RMSs compared with TD children during 500–700 ms, no significant differences were observed in any time-windows (P > 0.0031). In the right hemisphere, as shown in Fig 4D and 4E, although children with ASD tended to display lower RMSs compared with TD children during 200–600 ms, no significant differences were observed in any time-windows (P > 0.0031).

### AEF topography

**Fig 5:**
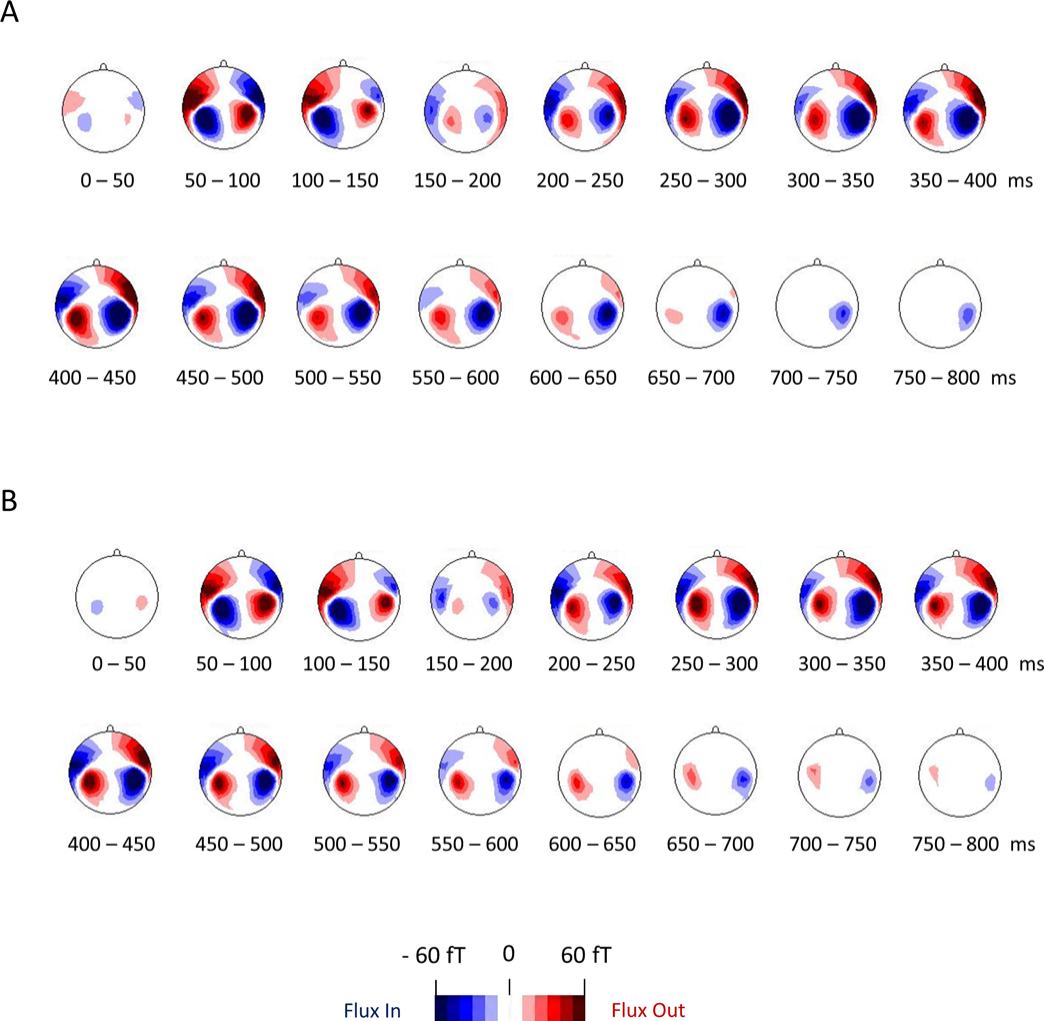
Magnitude topographies of whole-head AEFs for TD and ASD children. The magnitude topographies of the AEFs calculated for each 151-sensor (whole-head) examination of TD (A) and ASD children (B). For each 50 ms time window, an unpaired two-tailed *t*-test was used to compare the magnitudes observed for TD and ASD children. No significant differences between the two types of children were observed in any time window (P > 0.0031 for all comparisons). AEF, auditory evoked field. TD, typically developing. ASD, autism spectrum disorder.

As indicated in Fig 5, TD children (Fig 5A) and children with ASD (Fig 5B) exhibited similar AEF topographies. No significant difference in any sensor was observed in any time window (P > 0.0031 for all comparisons).

### Omega complexity analysis in AEFs for the whole head

**Fig 6:**
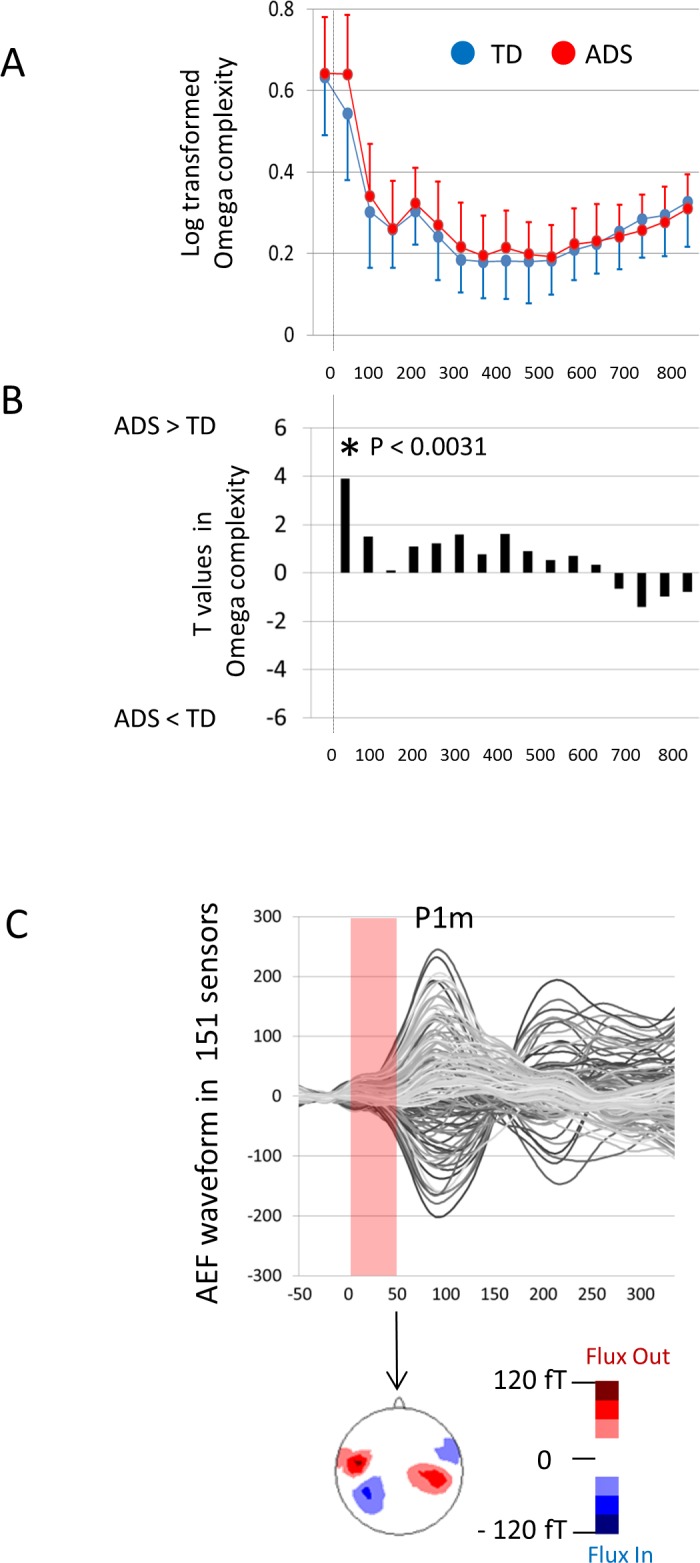
Omega complexity of AEFs for the whole head in TD and ASD children. (A) Omega complexities were calculated by 151 sensors (whole head) in TD and ASD children for each time-window of 50 milliseconds. (B) Log-10 transformed Omega complexities are compared between TD and ASD children with unpaired, two-tailed *t*-tests for each time window. Children with ASD exhibited significantly higher Omega complexities compared with TD children in the time-window of 0–50 ms (t = 3.90, *P* = 0.0002). (C) The time-window of 0–50 ms included the early stage of the P1m component. AEF, auditory evoked field. TD, typically developing. ASD, autism spectrum disorder. The error bars represent 1 standard deviation.

As shown in Fig 6A and 6B, children with ASD exhibited significantly higher Omega complexities compared with TD children in the time-window of 0–50 ms (t = 3.90, *P* = 0.0002). As shown in Fig 6C, the time-window of 0–50 ms where significance was observed included the beginning of the P1m component. No significant differences in any other time-windows were observed (P > 0.0031) (S1 Dataset).

As a complementary analysis considering the three distinct subtypes of ASD, one-way ANOVA of the four groups (i.e., TD, Asperger syndrome, and atypical and typical autism) was performed for the time-window of 0–50 ms, where significance was observed; there was a significant group effect (df = (3), F = 5.319, P = 0.0020). The post-hoc t-test revealed a significant difference only between the TD and typical autism groups (TD < typical autism; df = (81), t = 3.742, P = 0.0003), whereas no significant differences were found in other group pairs (P > 0.0083).

### Omega complexity in AEFs for the left and right hemisphere

**Fig 7:**
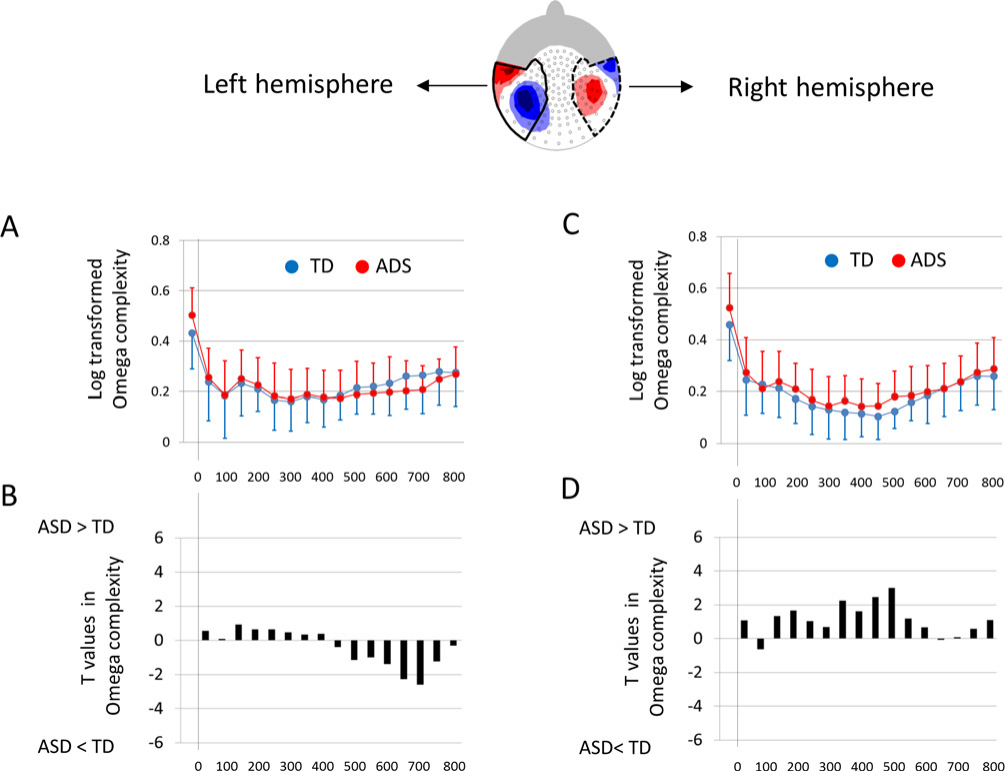
Omega complexity of AEFs for the left and right hemispheres in TD and ASD children. Omega complexities were calculated in 40 sensors corresponding to the left (A) and right (C) hemisphere. The Omega complexities of TD children and children with ASD were compared with unpaired two-tailed *t*-tests for 16 time windows in the left (B) and right (D) hemisphere. No significant differences were observed in any time-windows. AEF, auditory evoked field. TD, typically developing. ASD, autism spectrum disorder. The error bars represent 1 standard deviation.

In the left hemisphere, as shown in Fig 7A and 7B, although children with ASD tended to display lower Omega complexities compared with TD children during 500–700 ms, no significant differences were observed in any time-windows (P > 0.0031). In the right hemisphere, as shown in Fig 7C and 7D, although children with ASD tended to display higher Omega complexities compared with TD children during 200–500 ms, no significant differences in any time-windows were observed (P > 0.0031).

### Effects of age and cognitive ability on Omega complexity

**Fig 8:**
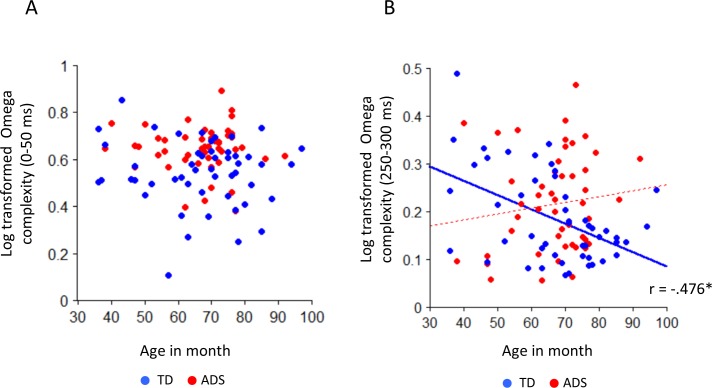
Scatter plot of log-transformed Omega complexity (250–300 ms) and age in months for ASD and TD children. (A) During the 0–50 ms time window, there was no significant correlation between Omega complexity and age in months for either TD children or children with ASD. (B) For TD children, a significant correlation between Omega complexity and age in months was only detected during the 250–300 ms time window. TD, typically developing. ASD, autism spectrum disorder. Solid blue line, regression line for TD children. Broken red line, regression line for ASD children. *, P < 0.0031.

As indicated in Table 2 and Fig 8B, age significantly affected Omega complexity in the 250–300 ms time window for TD children (P < 0.0031), and no significant correlations were observed in any other time window (P > 0.0031 for all windows) (Fig 8A). In both groups, no significant correlations between K-ABC mental processing scale score and Omega complexity were observed in any of the time windows.

**Table 2:** Pearson correlation coefficients between Omega complexity in AEFs for the whole head and either age or K-ABC mental processing scale score for children with ASD (n = 50) and TD children (n = 50).

Time windows (ms)	Age in months		K-ABC mental processing scale	
	ASD	TD	ASD	TD
0–50	n.s.	n.s.	n.s.	n.s.
50–100	n.s.	-.312	n.s.	-.402
100–150	n.s.	n.s.	n.s.	-.282
150–200	n.s.	n.s.	n.s.	n.s.
200–250	n.s.	n.s.	n.s.	n.s.
250–300	n.s.	-.476*	n.s.	n.s.
300–350	n.s.	-.309	n.s.	n.s.
350–400	n.s.	-.284	n.s.	n.s.
400–450	n.s.	-.349	n.s.	n.s.
450–500	n.s.	n.s.	n.s.	n.s.
500–550	n.s.	n.s.	n.s.	n.s.
550–600	n.s.	-.340	n.s.	n.s.
600–650	n.s.	-.330	n.s.	n.s.
650–700	n.s.	n.s.	n.s.	n.s.
700–750	n.s.	n.s.	n.s.	n.s.
750–800	n.s.	n.s.	n.s.	n.s.

n.s. = not significant. The r values are presented for P < 0.05.

*, significance at an alpha level of 0.0031. Please note that significance at an alpha level of 0.05 involves a risk of Type I error.

## Discussion

The present study is the first study to demonstrate atypical bilateral brain synchronization in the early stage of human voice auditory processing in young children with autism. Our custom-sized MEG system for children enables us to analyze bilateral AEFs simultaneously, which would otherwise be difficult to measure using conventional adult-sized MEG systems. Conventional whole-head MEG systems have fixed sensor arrays designed to accommodate most adult heads. However, arrays optimized for adult brain measurements are suboptimal for research with the significantly smaller heads of young children (i.e., the long distances between the brain and sensors considerably attenuate magnetic strengths). After the development of the child custom-sized MEG in 2010 [42], researchers could investigate whole brain activity in young children (e.g., 3–7 years old) [2,29,30,40,41,47,48], and the present study is the first to investigate interhemispheric brain synchronization in AEFs in young children with ASD.

As shown in Fig 6A and 6B, children with ASD exhibited significantly higher Omega complexities compared with TD children in the time-window of 0–50 ms, which suggests lower whole brain synchronization in the early stage of the P1m component. As indicated in Table 2, age and cognitive ability did not significantly affect bilateral brain synchronization in this time window. When we analyzed the left and right hemispheres separately, no significant differences between TD children and children with ASD were observed in any time-windows. These results suggest that the lower right-left hemispheric synchronization in children with ASD contributed to the observed lower whole brain synchronization in the early stage of the AEF component (i.e., higher Omega complexity in the whole brain). To investigate the magnitude of AEF that might affect Omega complexity, RMSs were calculated for the magnetic strengths recorded by 151 sensors for each time-window. No significant differences were observed between TD children and children with ASD. Furthermore, sensor-level (i.e., topographical) analysis also failed to demonstrate significant differences between TD children and children with ASD for any sensors in any time window. Therefore, irrespective of AEF strength, the complexity of 151-channel brain magnetic fields in the early stage of P1m is atypical in children with ASD.

Reports have indicated that the ASD population exhibits elevated rates of brain stem or peripheral hearing dysfunction [13–20] and abnormalities in AEF components (i.e., cortical dysfunction) [1–10,21–25]. Intriguingly, research has indicated that individuals with ASD may present with peripheral auditory asymmetry [13], which may distort the cortical bilateral synchronization of AEF components in the central nervous system. Reduced bilateral synchronization in the early stage of the P1m component in children with ASD may reflect peripheral auditory asymmetry and/or decreased interhemispheric cortical connectivity.

Decreased long-range cortical connectivity may also contribute to the lower bilateral synchronization in the early stage of the P1m component in children with ASD, because previous physiological studies suggested that within 40 ms after stimulus onset (before P1m peak), a sound already has an impact on neural activity in other brain regions (e.g., orofacial motor cortex) through the posterior lateral superior temporal and inferior frontal gyrus [49]. Time-windows of 0–50 ms may be not too early to reflect the brain bilateral cortical connectivity.

The main sources of the P1m and N1m are the auditory cortex and association cortices. These cortices process auditory information and are thought to function as a "semantic processor" to deduce the meaning of sounds via learning [50]. In these cortices, sensory bottom-up and attentional top-down processing are closely related [51,52]; therefore, in young children, dysfunction in this central system is thought to underlie various learning problems [53,54]. In the present study, although no children exhibited hearing loss, there were weak correlations between Omega complexity and general cognitive ability (|r| = 0.402) in the time window corresponding to the P1m peak (i.e., the 50–100 ms time window) in TD children. Therefore, lower bilateral synchronization in P1m may be a physiological marker of lower cognitive ability in TD young children but not in children with ASD. These results suggested that auditory cortices play different roles in (or have differing impacts on) cognitive development in TD children and in children with ASD.

As indicated in Fig 6A and 6B, children with ASD tended to exhibit higher Omega complexities than TD children in many time windows (such as time windows from 0 to 600 ms), which suggests that the former children exhibit reduced whole brain synchronization relative to the latter children. Our results are consistent with several recent reports of reduced brain hemodynamic synchronization in young children, adolescents and adults with ASD [55–59]. Two recent studies reported significantly decreased inter-hemispheric synchronization in toddlers with ASD [55] and in adolescents and adults with ASD [56]. Structural imaging has also demonstrated abnormalities in inter-hemispheric long-range white matter pathways. Corpus callosum volume has been used as an index of inter-hemispheric connectivity, and a reduced corpus callosum volume is one of the most replicated structural findings in ASD [60–65]. A diffusion tensor analysis of the corpus callosum also demonstrated significant microstructural differences between ASD and control groups [63,66–68]. Reduced inter-hemispheric connectivity might cause reduced inter-hemispheric synchronization during the auditory information processing observed in the present study.

Further research is necessary to reveal the symptomatological meaning of these reduced inter-hemispheric synchronizations in children with ASD.

## Limitations

The present study had some general limitations. First, we investigated AEFs using only one type of auditory stimulus (the human voice /ne/). Therefore, we cannot determine whether our results are specific to human voice stimuli. Second, a majority of the children with ASD examined in the present study were high-functioning subjects and therefore may not be representative of children with ASD who present with co-morbid language disabilities. Thus, the findings of this study may not apply to children with “Kanner's autism”. In addition, further study of each distinct subtype of ASD (i.e., Asperger syndrome, and atypical and typical autism) separately, using larger sample sizes, is necessary because these subtypes potentially result in different cognitive and language performances. Third, this investigation did not control for the socioeconomic statuses or intelligence levels of the examined parents and children. Fourth, we eliminated any contaminated MEG data, such as data obtained when clear head movement occurred. However, differences in the fine head movements of the children with ASD and the TD children could have confounded the study results. Fifth, we investigated AEFs with a short ISI (818 ms); for younger children, the duration of this ISI was insufficient to allow for a return to baseline from a stimulus. Therefore, the observed difference between TD children and children with ASD during an early time window (0–50 ms) could potentially have been caused by differences in sustained components of AEF activities; such differences could have been a confounding factor. Additional studies using longer ISIs are therefore necessary to reduce the effects of the sustained components of AEFs. Sixth, the observed differences in measured Omega complexity may have been related to the different spatiotemporal properties of the video stimuli provided during the recording periods. In addition, we did not evaluate the degree to which subjects focused on the TV program that they selected. Future studies that employ attention-controlled conditions will provide more reliable evidence than the current investigation, although such conditions will likely be difficult to achieve for conscious preschool-aged children.

## Supporting Information

S1 Dataset This dataset contains individual Log-10 transformed Omega complexities calculated by 151 sensors (whole head) for each time-window of 50 milliseconds. (XLSX)

S1 Fig The performance (raw score) of each Kaufman Assessment Battery (K-ABC) subtest in children with ASD and TD young children is shown. (DOC)

## References

[1] Edgar JC, Lanza MR, Daina AB, Monroe JF, Khan SY, Blaskey L, et al Missing and delayed auditory responses in young and older children with autism spectrum disorders. Front Hum Neurosci. 2014;8:417 10.3389/fnhum.2014.00417 24936181 PMC4047517

[2] Yoshimura Y, Kikuchi M, Shitamichi K, Ueno S, Munesue T, Ono Y, et al Atypical brain lateralisation in the auditory cortex and language performance in 3- to 7-year-old children with high-functioning autism spectrum disorder: a child-customised magnetoencephalography (MEG) study. Mol Autism. 2013;4:38 10.1186/2040-2392-4-38 24103585 PMC4021603

[3] Roberts TP, Cannon KM, Tavabi K, Blaskey L, Khan SY, Monroe JF, et al Auditory Magnetic Mismatch Field Latency: A Biomarker for Language Impairment in Autism. Biol Psychiatry. 2011. doi: S0006-3223(11)00063-1. 10.1016/j.biopsych.2011.01.015 PMC3134608 21392733

[4] Roberts TP, Khan SY, Rey M, Monroe JF, Cannon K, Blaskey L, et al MEG detection of delayed auditory evoked responses in autism spectrum disorders: towards an imaging biomarker for autism. Autism Res. 2010;3:8–18. 10.1002/aur.111 20063319 PMC3099241

[5] Schmidt GL, Rey MM, Oram Cardy JE, Roberts TP. Absence of M100 source asymmetry in autism associated with language functioning. Neuroreport. 2009;20:1037–1041. 10.1097/WNR.0b013e32832e0ca7 19491710 PMC2795634

[6] Flagg EJ, Cardy JE, Roberts W, Roberts TP. Language lateralization development in children with autism: insights from the late field magnetoencephalogram. Neurosci Lett. 2005;386:82–87. doi: S0304-3940(05)00583-5. 16046066 10.1016/j.neulet.2005.05.037

[7] Oram Cardy JE, Flagg EJ, Roberts W, Roberts TP. Delayed mismatch field for speech and non-speech sounds in children with autism. Neuroreport. 2005;16:521–525. 15770164 10.1097/00001756-200504040-00021

[8] Oram Cardy JE, Flagg EJ, Roberts W, Brian J, Roberts TP. Magnetoencephalography identifies rapid temporal processing deficit in autism and language impairment. Neuroreport. 2005;16:329–332. doi: 00001756-200503150-00005. 15729132 10.1097/00001756-200503150-00005

[9] Matsuzaki J, Kagitani-Shimono K, Goto T, Sanefuji W, Yamamoto T, Sakai S, et al Differential responses of primary auditory cortex in autistic spectrum disorder with auditory hypersensitivity. Neuroreport. 2012;23:113–118. 10.1097/WNR.0b013e32834ebf44 22146579

[10] Matsuzaki J, Kagitani-Shimono K, Sugata H, Hirata M, Hanaie R, Nagatani F, et al Progressively increased M50 responses to repeated sounds in autism spectrum disorder with auditory hypersensitivity: a magnetoencephalographic study. PLoS One. 2014;9:e102599 10.1371/journal.pone.0102599 25054201 PMC4108353

[11] Orekhova EV, Tsetlin MM, Butorina AV, Novikova SI, Gratchev VV, Sokolov PA, et al Auditory cortex responses to clicks and sensory modulation difficulties in children with autism spectrum disorders (ASD). PLoS One. 2012;7:e39906 10.1371/journal.pone.0039906 22768163 PMC3387220

[12] Wilson TW, Rojas DC, Reite ML, Teale PD, Rogers SJ. Children and adolescents with autism exhibit reduced MEG steady-state gamma responses. Biol Psychiat. 2007;62:192–197. 10.1016/j.biopsych.2007.07.002 16950225 PMC2692734

[13] Khalfa S, Bruneau N, Roge B, Georgieff N, Veuillet E, Adrien JL, et al Peripheral auditory asymmetry in infantile autism. Eur J Neurosci. 2001;13:628–632. 11168571 10.1046/j.1460-9568.2001.01423.x

[14] Demopoulos C, Lewine JD. Audiometric Profiles in Autism Spectrum Disorders: Does Subclinical Hearing Loss Impact Communication? Autism Res. 2015 10.1002/aur.1495 PMC4641833 25962745

[15] Roth DA, Muchnik C, Shabtai E, Hildesheimer M, Henkin Y. Evidence for atypical auditory brainstem responses in young children with suspected autism spectrum disorders. Dev Med Child Neurol. 2012;54:23–29. 10.1111/j.1469-8749.2011.04149.x 22142282

[16] Hitoglou M, Ververi A, Antoniadis A, Zafeiriou DI. Childhood Autism and Auditory System Abnormalities. Pediatr Neurol. 2010;42:309–314. 10.1016/j.pediatrneurol.2009.10.009 20399382

[17] Rosenhall U, Nordin V, Brantberg K, Gillberg C. Autism and auditory brain stem responses. Ear Hear. 2003;24:206–214. 10.1097/01.AUD.0000069326.11466.7E 12799542

[18] Skoff BF, Mirsky AF, Turner D. Prolonged brainstem transmission time in autism. Psychiatry Res. 1980;2:157–166. 6251502 10.1016/0165-1781(80)90072-4

[19] Klin A. Auditory brainstem responses in autism: brainstem dysfunction or peripheral hearing loss? J Autism Dev Disord. 1993;23:15–35. 8463195 10.1007/BF01066416

[20] Tanguay PE, Edwards RM, Buchwald J, Schwafel J, Allen V. Auditory brainstem evoked responses in autistic children. Arch Gen Psychiatry. 1982;39:174–180. 6279048 10.1001/archpsyc.1982.04290020040008

[21] Roberts TP, Lanza MR, Dell J, Qasmieh S, Hines K, Blaskey L, et al Maturational differences in thalamocortical white matter microstructure and auditory evoked response latencies in autism spectrum disorders. Brain Res. 2013;1537:79–85. 10.1016/j.brainres.2013.09.011 24055954 PMC3970268

[22] Brock J, Bzishvili S, Reid M, Hautus M, Johnson BW. Brief report: atypical neuromagnetic responses to illusory auditory pitch in children with autism spectrum disorders. J Autism Dev Disord. 2013;43:2726–2731. 10.1007/s10803-013-1805-z 23543291

[23] Oram Cardy JE, Ferrari P, Flagg EJ, Roberts W, Roberts TP. Prominence of M50 auditory evoked response over M100 in childhood and autism. Neuroreport. 2004;15:1867–1870. doi: 00001756-200408260-00006. 15305126 10.1097/00001756-200408260-00006

[24] Gage NM, Siegel B, Callen M, Roberts TP. Cortical sound processing in children with autism disorder: an MEG investigation. Neuroreport. 2003;14:2047–2051. 10.1097/01.wnr.0000090030.46087.4a 14600495

[25] Gage NM, Siegel B, Roberts TP. Cortical auditory system maturational abnormalities in children with autism disorder: an MEG investigation. Brain Res Dev Brain Res. 2003;144:201–209. doi: S016538060300172X. 12935917 10.1016/s0165-3806(03)00172-x

[26] Ghanbari Y, Bloy L, Batmanghelich K, Roberts TP, Verma R. Dominant component analysis of electrophysiological connectivity networks. Medical image computing and computer-assisted intervention: MICCAI International Conference on Medical Image Computing and Computer-Assisted Intervention. 2012;15:231–238. 10.1007/978-3-642-33454-2_29 PMC4029114 23286135

[27] Hiraishi H, Kikuchi M, Yoshimura Y, Kitagawa S, Hasegawa C, Munesue T, et al Unusual developmental pattern of brain lateralization in young boys with autism spectrum disorder: Power analysis with child-sized magnetoencephalography. Psychiatry Clin Neurosci. 2015;69:153–160. 10.1111/pcn.12261 25439739

[28] Kikuchi M, Yoshimura Y, Hiraishi H, Munesue T, Hashimoto T, Tsubokawa T, et al Reduced long-range functional connectivity in young children with autism spectrum disorder. Social cognitive and affective neuroscience. 2015;10:248–254. 10.1093/scan/nsu049 24652855 PMC4321624

[29] Kikuchi M, Shitamichi K, Yoshimura Y, Ueno S, Hiraishi H, Hirosawa T, et al Altered brain connectivity in 3-to 7-year-old children with autism spectrum disorder. NeuroImage: Clinical. 2013; 2:394–401. 24179793 10.1016/j.nicl.2013.03.003 PMC3777701

[30] Kikuchi M, Yoshimura Y, Shitamichi K, Ueno S, Hirosawa T, Munesue T, et al A custom magnetoencephalography device reveals brain connectivity and high reading/decoding ability in children with autism. Sci Rep. 2013;3:1139 10.1038/srep01139 23355952 PMC3555087

[31] Sharma A, Kraus N, McGee TJ, Nicol TG. Developmental changes in P1 and N1 central auditory responses elicited by consonant-vowel syllables. Electroencephalogr Clin Neurophysiol. 1997;104:540–545. 9402896 10.1016/s0168-5597(97)00050-6

[32] Ponton C, Eggermont JJ, Khosla D, Kwong B, Don M. Maturation of human central auditory system activity: separating auditory evoked potentials by dipole source modeling. Clin Neurophysiol. 2002;113:407–420. doi: S1388245701007337. 11897541 10.1016/s1388-2457(01)00733-7

[33] Gilley PM, Sharma A, Dorman M, Martin K. Developmental changes in refractoriness of the cortical auditory evoked potential. Clin Neurophysiol. 2005;116:648–657. doi: S1388-2457(04)00367-0. 15721079 10.1016/j.clinph.2004.09.009

[34] Wackermann J. Beyond mapping: estimating complexity of multichannel EEG recordings. Acta Neurobiol Exp (Wars). 1996;56:197–208. 8787174 10.55782/ane-1996-1121

[35] Wackermann J. Towards a quantitative characterisation of functional states of the brain: from the non-linear methodology to the global linear description. Int J Psychophysiol. 1999;34:65–80. 10555875 10.1016/s0167-8760(99)00038-0

[36] Lord C, Rutter M, DiLavore P, Risi S. Autism Diagnostic Observation Schedule. Los Angeles, CA: Western Psychological Services; 1999.

[37] Wing L, Leekam SR, Libby SJ, Gould J, Larcombe M. The Diagnostic Interview for Social and Communication Disorders: background, inter-rater reliability and clinical use. J Child Psychol Psychiatry. 2002;43:307–325. 11944874 10.1111/1469-7610.00023

[38] Kaufman A, Kaufman N. Kaufman Assessment Battery for Children: Administration and Scoring Manual Circle Pines. Minnesota: American Guidance Service; 1983.

[39] American Psychiatric Association. Diagnostic and Statistical Manual of Mental Disorders (DSM-IV). Washington D.C.: Amer Psychiatric Pub; 1994.

[40] Yoshimura Y, Kikuchi M, Shitamichi K, Ueno S, Remijn GB, Haruta Y, et al Language performance and auditory evoked fields in 2- to 5-year-old children. Eur J Neurosci. 2012;35:644–650. 10.1111/j.1460-9568.2012.07998.x 22321133

[41] Yoshimura Y, Kikuchi M, Ueno S, Shitamichi K, Remijn GB, Hiraishi H, et al A longitudinal study of auditory evoked field and language development in young children. Neuroimage. 2014;101:440–447. 10.1016/j.neuroimage.2014.07.034 25067819

[42] Johnson BW, Crain S, Thornton R, Tesan G, Reid M. Measurement of brain function in pre-school children using a custom sized whole-head MEG sensor array. Clin Neurophysiol. 2010;121:340–349. 10.1016/j.clinph.2009.10.017 19955015

[43] Cook HM. The sentence-final particle ne as a tool for cooperation in Japanese convcrsation. Thc Stanford Linguistic Association, Stanford 1990.

[44] Anderson V, Hiramoto M, Wong A. Prosodic Analysis of the Interactional Particle Ne in Japanese Gendered Speech. Japanese/Korean Linguistics. 2007;15:43–54.

[45] Kajikawa S, Amano S, Kondo T. Speech overlap in Japanese mother-child conversations. J Child Lang. 2004;31:215–230. 15053091

[46] Squires T. A discourse Anlysis of the Japanese Particle sa. Pragmatics. 2009;4:1–29.

[47] Kikuchi M, Shitamichi K, Yoshimura Y, Ueno S, Remijn GB, Hirosawa T, et al Lateralized theta wave connectivity and language performance in 2- to 5-year-old children. J Neurosci. 2011;31:14984–14988. 10.1523/JNEUROSCI.2785-11.2011 22016531 PMC6623588

[48] Remijn GB, Kikuchi M, Shitamichi K, Ueno S, Yoshimura Y, Nagao K, et al Somatosensory evoked field in response to visuotactile stimulation in 3- to 4-year-old children. Front Hum Neurosci. 2014;8:170 10.3389/fnhum.2014.00170 24715860 PMC3970025

[49] Patterson RD, Johnsrude IS. Functional imaging of the auditory processing applied to speech sounds. Philos Trans R Soc Lond B Biol Sci. 2008;363:1023–1035. doi: P222761575PTM361. 17827103 10.1098/rstb.2007.2157 PMC2606794

[50] Scheich H, Brechmann A, Brosch M, Budinger E, Ohl FW, Selezneva E, et al Behavioral semantics of learning and crossmodal processing in auditory cortex: the semantic processor concept. Hear Res. 2011;271:3–15. 10.1016/j.heares.2010.10.006 20971178

[51] Schadow J, Lenz D, Dettler N, Frund I, Herrmann CS. Early gamma-band responses reflect anticipatory top-down modulation in the auditory cortex. Neuroimage. 2009;47:651–658. 10.1016/j.neuroimage.2009.04.074 19414068

[52] Bailey T. Beyond DSM: the role of auditory processing in attention and its disorders. Applied neuropsychology Child. 2012;1:112–120. 10.1080/21622965.2012.703890 23428298

[53] Cacace AT, McFarland DJ. Central auditory processing disorder in school-aged children: a critical review. J Speech Lang Hear Res. 1998;41:355–373. 9570588 10.1044/jslhr.4102.355

[54] Seither-Preisler A, Parncutt R, Schneider P. Size and synchronization of auditory cortex promotes musical, literacy, and attentional skills in children. J Neurosci. 2014;34:10937–10949. 10.1523/JNEUROSCI.5315-13.2014 25122894 PMC6705250

[55] Dinstein I, Pierce K, Eyler L, Solso S, Malach R, Behrmann M, et al Disrupted neural synchronization in toddlers with autism. Neuron. 2011;70:1218–1225. 10.1016/j.neuron.2011.04.018 21689606 PMC3119852

[56] Anderson JS, Druzgal TJ, Froehlich A, DuBray MB, Lange N, Alexander AL, et al Decreased interhemispheric functional connectivity in autism. Cereb Cortex. 2011;21:1134–1146. 10.1093/cercor/bhq190 20943668 PMC3077433

[57] Cherkassky VL, Kana RK, Keller TA, Just MA. Functional connectivity in a baseline resting-state network in autism. Neuroreport. 2006;17:1687–1690. 10.1097/01.wnr.0000239956.45448.4c 17047454

[58] Kennedy DP, Courchesne E. The intrinsic functional organization of the brain is altered in autism. Neuroimage. 2008;39:1877–1885. 10.1016/j.neuroimage.2007.10.052 18083565

[59] Monk CS, Peltier SJ, Wiggins JL, Weng SJ, Carrasco M, Risi S, et al Abnormalities of intrinsic functional connectivity in autism spectrum disorders. Neuroimage. 2009;47:764–772. 10.1016/j.neuroimage.2009.04.069 19409498 PMC2731579

[60] Manes F, Piven J, Vrancic D, Nanclares V, Plebst C, Starkstein SE. An MRI study of the corpus callosum and cerebellum in mentally retarded autistic individuals. J Neuropsychiatry Clin Neurosci. 1999;11:470–474. 10570760 10.1176/jnp.11.4.470

[61] Hardan AY, Minshew NJ, Keshavan MS. Corpus callosum size in autism. Neurology. 2000;55:1033–1036. 11061265 10.1212/wnl.55.7.1033

[62] Vidal CN, Nicolson R, DeVito TJ, Hayashi KM, Geaga JA, Drost DJ, et al Mapping corpus callosum deficits in autism: an index of aberrant cortical connectivity. Biol Psychiatry. 2006;60:218–225. 10.1016/j.biopsych.2005.11.011 16460701

[63] Alexander AL, Lee JE, Lazar M, Boudos R, DuBray MB, Oakes TR, et al Diffusion tensor imaging of the corpus callosum in Autism. Neuroimage. 2007;34:61–73. doi: S1053-8119(06)00890-1. 17023185 10.1016/j.neuroimage.2006.08.032

[64] Stanfield AC, McIntosh AM, Spencer MD, Philip R, Gaur S, Lawrie SM. Towards a neuroanatomy of autism: a systematic review and meta-analysis of structural magnetic resonance imaging studies. European psychiatry: the journal of the Association of European Psychiatrists. 2008;23:289–299. 10.1016/j.eurpsy.2007.05.006 17765485

[65] Keary CJ, Minshew NJ, Bansal R, Goradia D, Fedorov S, Keshavan MS, et al Corpus callosum volume and neurocognition in autism. J Autism Dev Disord. 2009;39:834–841. 10.1007/s10803-009-0689-4 19165587 PMC3229274

[66] Wolff JJ, Gu H, Gerig G, Elison JT, Styner M, Gouttard S, et al Differences in white matter fiber tract development present from 6 to 24 months in infants with autism. Am J Psychiatry. 2012;169:589–600. 10.1176/appi.ajp.2011.11091447 22362397 PMC3377782

[67] Keller TA, Kana RK, Just MA. A developmental study of the structural integrity of white matter in autism. Neuroreport. 2007;18:23–27. 10.1097/01.wnr.0000239965.21685.99 17259855

[68] Brito AR, Vasconcelos MM, Domingues RC, Hygino da Cruz LC Jr., Rodrigues Lde S, Gasparetto EL, et al Diffusion tensor imaging findings in school-aged autistic children. Journal of neuroimaging: official journal of the American Society of Neuroimaging. 2009;19:337–343. 10.1111/j.1552-6569.2009.00366.x 19490374

