# Supplementary material for: Atypical Bilateral Brain Synchronization in the Early Stage of Human Voice Auditory Processing in Young Children with Autism
Source: PLoS One. 2016 Apr 13;11(4):e0153077. doi: 10.1371/journal.pone.0153077 (PMC4830448; doi:10.1371/journal.pone.0153077)
Supplement: S1 Fig — (DOC) [file pone.0153077.s002.doc]

Plos One

**Supplemental information**

**Title: Atypical bilateral brain synchronization in the early stage of human voice auditory processing in young children with autism**

**S1 Fig
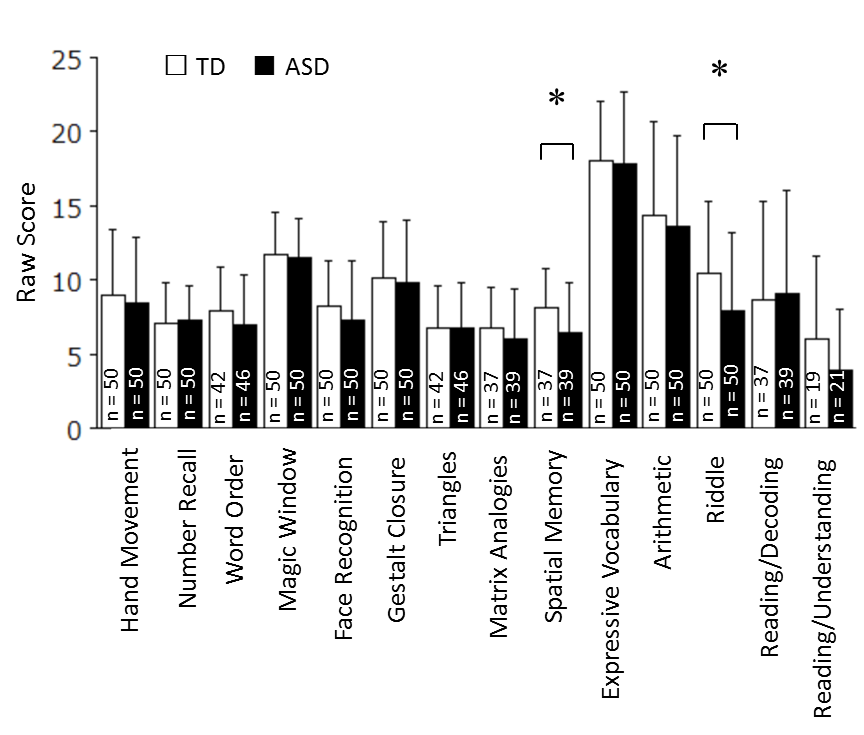
**

S1 Fig. The performance (raw score) of each Kaufman Assessment Battery (K-ABC) subtest in children with ASD and TD young children is shown. The error bars represent 1 standard deviation. An unpaired *t*-test revealed a significantly lower performance in children with ASD compared with TD children in two subtests (i.e., “Spatial Memory” and “Riddle” subtests). “Spatial Memory” subtest reflects visual working memory and “Riddle” subtest reflects language conceptual inference ability. K-ABC is typically used to assess the cognitive skills of children aged 30-155 months. To confirm the cognitive scales in children, subtests that were complementary to the age (in months) of the children were used in this battery. Each subtest has suitable age range, and therefore, sample size was different among subtests. **P*<0.05.
